# Distribution of bladder afferent activity across the sacral roots in sheep shows marked individual variation: implications for neuroprosthesis design

**DOI:** 10.3389/fnins.2026.1857570

**Published:** 2026-06-12

**Authors:** Jon Prager, Maria Petrou, Martin Leonhardt, Leen Jabban, Nicolas Granger, John Taylor, Nick Donaldson, Benjamin Metcalfe

**Affiliations:** 1 Clinical Science and Services, Royal Veterinary College, Hatfield, United Kingdom; 2 Department of Electronic and Electrical Engineering, University of Bath, Bath, United Kingdom; 3 Bath Institute for the Augmented Human, University of Bath, Bath, United Kingdom; 4 Department of Medical Physics and Biomedical Engineering, University College London, London, United Kingdom

**Keywords:** bladder afferent ENG, Brindley device, neuroprosthesis, sacral anterior root stimulation, sacral roots, urinary bladder pressure

## Abstract

**Objective:**

Implantable sacral anterior root stimulators enable bladder emptying after spinal cord injury but do not prevent reflex incontinence. A closed-loop neuroprosthesis that detects and inhibits reflex bladder contractions could address this, but first, reliable detection of bladder fullness from the sacral roots. Further, the distribution of afferent bladder activity between sacral roots, and the relationship between efferent and afferent activity within each root, remains unclear and must be clarified to guide implant design.

**Methods:**

Electrode books were implanted on the S1–S3 extra-dural sacral roots bilaterally in six terminally anesthetized sheep. Afferent electroneurogram (ENG) was recorded concurrently from all implanted roots during filling cystometries and correlated with bladder pressure. Each root was individually electrically stimulated and the bladder pressure response recorded. Post-mortem morphometric analysis determined fiber size distribution in each root.

**Results:**

Overall, S2 ENG activity showed the highest correlation with bladder pressure, and electrical stimulation of S2 and S3 produced the greatest increases in bladder pressure. Fiber size distribution did not correlate with either ENG activity or bladder pressure response. Significant variation was identified between individual sheep, but notably, in four of six sheep, a single sacral root had both the highest ENG correlation to bladder pressure and the greatest bladder response to stimulation.

**Significance:**

This study demonstrates reliable recording of bladder afferents from sacral roots using clinically applicable electrodes. It provides the first systematic recording of bladder ENG concurrently across three pairs of sacral roots in multiple animals, and the first characterization of signal distribution between roots. Significant individual variation is identified, impacting the design of future implantable sacral neuroprostheses for bladder control.

## 1 Introduction

Dependent on the location and severity of damage, spinal cord injury (SCI) can cause chronic and irreversible urinary incontinence with dysfunction in both storage and voiding of urine. This has a major impact on quality of life for patients; return of bladder and bowel control is valued more highly than return of walking for people with thoracic and thoracolumbar SCI (Liu et al., 2010; Anderson, 2004).

In supra-sacral SCI, the motor neuron innervation to the bladder remains intact, but the disruption or absence of descending input prevents conscious control of voiding, which would normally trigger synchronous contraction of the detrusor muscle and relaxation of the urethral sphincter. Reflex bladder activity remains, however, resulting in detrusor contraction against a closed urethral sphincter (detrusor-sphincter dyssynergia, DSD), increasing bladder pressures and causing intermittent urinary leakage.

Increased bladder pressures can cause autonomic dysreflexia or hydronephrosis and ultimately renal failure, with significant morbidity and mortality. To manage bladder fullness and reduce reflex incontinence, most patients must self-catheterise multiple times a day in the chronic phase after injury. However, this is invasive, expensive and infection-prone (Chamberlain et al., 2019; Milligan et al., 2020).

A clinically employed alternative, the implantable Finetech-Brindley Sacral Anterior Root Stimulator (SARS), allows patients to selectively void their bladder (Brindley and Frs, 1994). Intermittent stimulation of the sacral anterior roots induces co-contraction of the detrusor and urethral sphincter, followed by a post-stimulation interval during which the urethral sphincter striated muscle relaxes more rapidly than the detrusor smooth muscle, creating a window for urine flow. However, to improve bladder storage and reduce reflex bladder contractions, surgeons typically perform a posterior rhizotomy, which interrupts the afferent limb of sacral reflex circuits. While this reduces reflex bladder contractions and incontinence, this procedure is undesirable as it is irreversible and removes reflexive functions, including sexual responses.

Detecting bladder pressure from the sacral roots might enable the design of a closed-loop-control neuroprosthesis for the management of bladder function after SCI without the need for rhizotomy. Proof-of-principle has been demonstrated in rats where bladder pressure was detected from dorsal sacral roots teased out into microchannels, and high frequency stimulation of the ventral roots blocked bladder contractions (Chew et al., 2013). Further proof of principle of this approach has been demonstrated in cats, using microelectrode arrays in the sacral dorsal root ganglion to record afferent bladder activity (Ouyang et al., 2022).

Neural signals encoding bladder fullness can also be recorded from the sacral roots using cuff electrodes, and this has been shown in pigs (Jezernik et al., 2000), cats (Jezernik et al., 2001), and sheep (Metcalfe et al., 2020). Recording from the pelvic nerve is also possible (Jezernik et al., 2000). Although the pelvic nerve showed a greater signal amplitude, this site is more difficult to access in humans; in contrast, the safety of surgical access and implantation on the sacral roots is well-characterized by the Finetech-Brindley SARS.

To minimize iatrogenic damage, surgical time, and infection risk, it would be desirable to implant a sensory device on as few roots as is necessary to determine bladder pressure reliably. Anatomical and electrophysiological studies have shown that afferent information on bladder fullness is transmitted in the pelvic and pudendal nerves via the sacral dorsal root ganglia to the spinal cord, primarily by Aδ myelinated axons responding to mucosal stretch. Unmyelinated C fibers generally respond to noxious stimuli, although they may also respond to volume, particularly at higher pressures (for reviews see de Groat et al., 2015; Yoshimura and Chancellor, 2003). There is also afferent information from the bladder in the hypogastric nerve, which arises from thoracolumbar segments.

In humans, the pudendal nerve arises predominantly from the S2 and S3 roots, with some contribution from S1 and S4 roots, and with individual asymmetry and variation (Vodušek, 2004). The nerve travels from the pelvic plexus through the greater sciatic foramen of the pelvis to innervate the gluteal region, pudendal canal and perineum, including the external genitalia, bladder and rectal sphincters (Possover and Forman, 2012). The route of the pelvic splanchnic nerves is more complex, involving the pelvic plexus (also known as the inferior hypogastric plexus), but also arises primarily from S2 to S4, with around 18% of women having additional innervation to S1 (Aurore et al., 2020).

Electrical stimulation of the sacral roots, one by one, while observing bladder pressure, gives a direct functional indication of the distribution of efferent innervation to the bladder, and is used to select the roots for SARS implantation. The greatest bladder contraction in people is most commonly seen in response to S3, with some input from S2 and S4 (Brindley et al., 1982; Rijkhoff et al., 1998; Dai and Xiao, 2005). It is worth noting there may be differences between species, relevant to the interpretation of data from animal models (Granger et al., 2013; Koldewijn et al., 1994; Sievert et al., 2002; Martinez-Pineiro et al., 1992).

Importantly, it is not currently clear how bladder afferents might be distributed between the sacral roots and whether efferent and afferent innervation are matched in the extra-dural sacral roots in a given individual. Therefore, to determine the functional distribution of bladder afferents in the sacral roots and to guide the design of a sensory device, a series of acute experiments in sheep was conducted. The aims of these experiments were to:

(i) Simultaneously record electroneurogram via electrode books from the extra-dural S1–S3 roots concurrently while performing cystometry;
(ii) Record bladder response to electrical stimulation of the sacral roots in these same sheep; and
(iii) Assess the distribution of fiber diameters within each root using morphometry.

It was hypothesized that afferent activity (ENG activity during cystometry) and efferent activity (bladder response to electrical stimulation) would be highest in the same root, but that this root would vary between individuals. A further hypothesis was that fiber size distribution would reflect this variation between individuals, with more fibers of a size consistent with afferent innervation found in the root(s) with the highest afferent activity.

This study, therefore, investigates the feasibility of concurrent bladder ENG recording across multiple sacral roots using clinically applicable electrode books and assesses the distribution of activity across roots. Additionally, a comparison is made among ENG correlation with bladder pressure, bladder pressure changes in response to electrical stimulation of the sacral roots, and fiber size distribution within the sacral roots.

## 2 Methods

To evaluate the relationship between concurrent bladder ENG recordings across multiple sacral roots, each experiment began with preoperative cystometry to establish bladder pressure baselines. A surgical approach to the sacral roots was then performed and electrical stimulation applied, followed by surgical implantation of electrode books on the bilateral S1–S3 sacral roots. Impedance measurements were taken to assess electrode integrity before connecting the implants to custom bipolar amplifiers for ENG recording. ENG recordings were performed across multiple cystometries. Animals were then terminated, and the roots were sampled for morphometric analysis. Signal-processing analyses were performed to assess the afferent distribution and the ENG correlation with bladder pressures.

### 2.1 Electrode book construction and wiring

**Figure 1:**
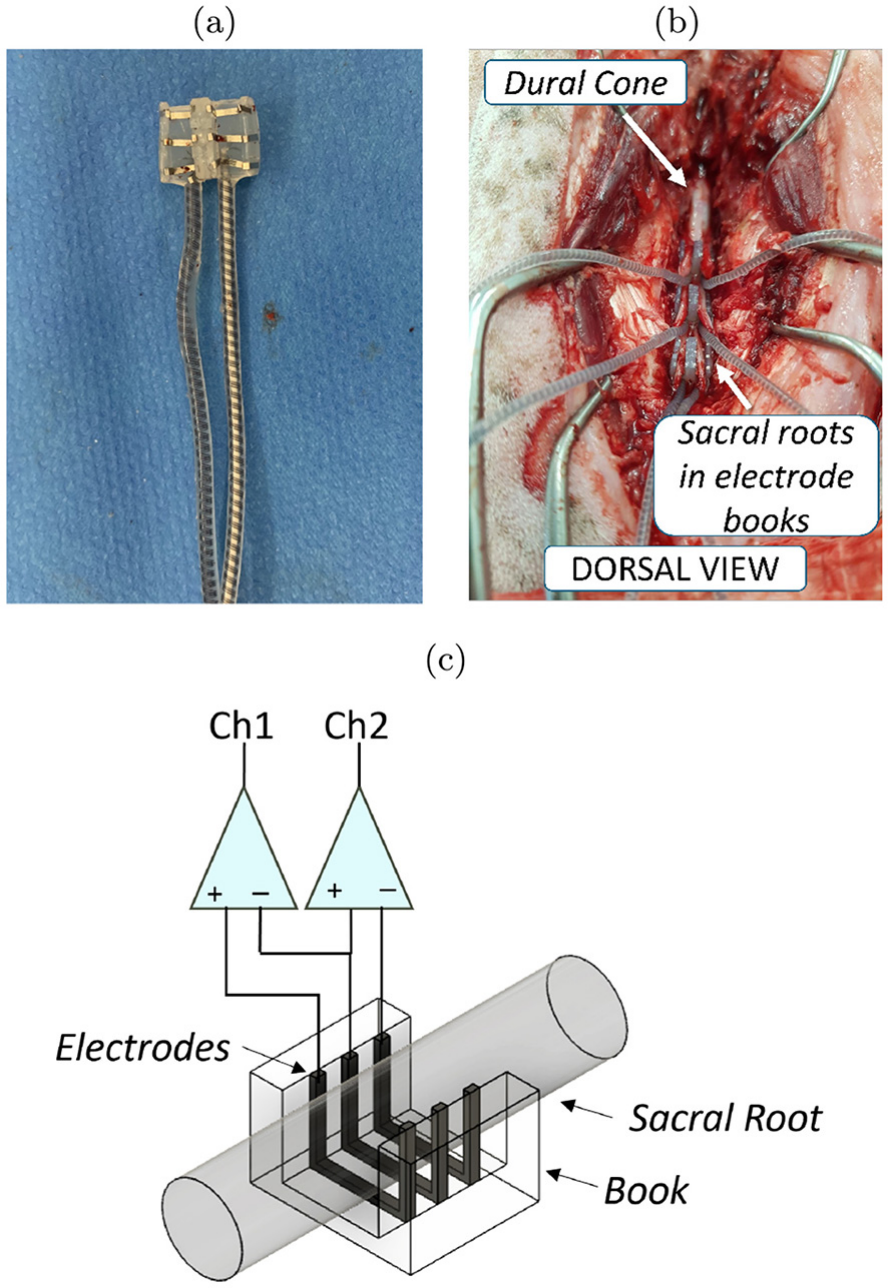
Images of electrode book **(a)** and electrode books *in situ* **(b)**. Note the three “U"-shaped platinum-iridium electrodes on each side. The sacral roots are labeled within the electrode books in **(b)**. Channels 1 and 2 of each book as in **(c)**.

Open, dual-cavity cuff electrodes, commonly referred to as electrode books, were custom-made by Finetech Medical (UK). Each consisted of two slots side-by-side, each with silicone insulating material and three “U"-shaped platinum-iridium electrodes with electrode spacing of 3 mm, width of 11 mm, height of 7 mm and a pitch of 3.5 mm (Figure 1A). One outer side was lowered to half the height to enable easier placement of sacral roots (Figure 1B). Each book was wired to two mini-XLR plugs via two 3-core Cooper cables (Carrington et al., 2005), one for each slot, with each slot connected as two bipoles (referred to as channels 1 and 2, Figure 1C).

### 2.2 Animal use and experimental design

Acute experiments were performed in six adult female mule sheep under terminal general anesthesia, with mean weights 61.5 ± 15.6 kg. *In vivo* experiments were conducted with local institutional ethical permission [RVC AWERB (Animal Welfare and Ethical Review Board)] and under the UK Animal (Scientific Procedures) Act (project license: P3FB23631). Reporting is according to ARRIVE guidelines. Before the experiments, the sheep were group-housed in pastures or large straw pens, with ad lib food and water.

### 2.3 Cystometry

As previously described, sheep received a transdermal fentanyl patch 24 h before surgery and were starved overnight prior to induction of anesthesia (Prager et al., 2024). Anesthesia was induced with intravenous ketamine (7.5 mg/kg) and midazolam (0.5 mg/kg) and maintained after intubation with sevoflurane in oxygen. They were ventilated, and standard physiological parameters were monitored and controlled within normal limits, including heart rate, respiratory rate, end-tidal CO_2_, SpO_2_, arterial blood pressure, electrocardiogram and temperature. We used a clinically applicable anesthetic protocol, rather than commonly used neurophysiology experiment anesthetic protocols involving alpha-chloralose or urethane, with the rationale that any further clinical translation of this work would likely need initial implantation and testing under anesthesia, and therefore, the system needs to be robust enough to detect afferent signals in this situation.

Repeat cystometries were performed in each sheep. An 8 French urethral 2-channel catheter connected to a commercial system (Medica PicoSmart) was used to fill the bladder with room-temperature sterile saline at a rate of 100 ml/min and measure intravesicular pressure, zeroed at the level of the urethral meatus for reference. A 12 French Foley catheter with a 10 ml balloon was also placed and used to prevent passive saline outflow from the bladder. The bladder was filled to 50 cmH_2_O or until leaking occurred.

### 2.4 Surgical approach and electrical stimulation of the sacral roots

A midline incision was made over the vertebral column from L6 to the caudal sacrum. Musculature was reflected off the median sacral crest, and a dorsal laminectomy was performed through the sacrum from the level of S1–S4. The S1 root was identified by size, anatomical path and position, then the bone window was widened until the electrode books could fit in the channel. S4 was inconsistently identified in the initial experiments; therefore, electrodes were implanted only in the first three sacral roots (S1–S3).

Prior to implantation of the electrode books, the bladder was filled to 20 cmH_2_O, and each S1–S3 root was electrically stimulated, in turn, using bipolar extra-dural hook electrodes and a clinically available surgical stimulator (Finetech Medical BSC259 and BSD260). Stimulation was performed at 30 Hz, rapidly ramping from 1 to 10 V and holding at 10 V for 5 s, using a monophasic square-wave pulse waveform with a pulse duration of 350 μs and a pulse frequency of 3 Hz. Peak bladder pressure after stimulation was recorded, and the increase in bladder pressure from baseline was calculated to assess bladder response to electrical stimulation of each sacral root.

Bilateral S1–S3 roots were subsequently encircled in sterile nylon tape and gently lifted into each side of the electrode book in turn (Figure 1B), working caudal to cranial. Kwik-sil (World Precision Instruments: 175 Sarasota Center Blvd, Sarasota, FL 34240. https://wpiinc.com/?srsltid=AfmBOoqYoPWd3WnkZOm0SR2a-0k9bOyyHS4NyFq4LyPf_O4JNJilBAep) was used to seal each root in its electrode.

### 2.5 Impedance measures

Two-wire impedance measurements were obtained between electrodes 1–2 and 2–3 bilaterally (channels 1 and 2, Figure 1C) on each book using a Keysight LCR meter at 100 Hz, 1 kHz, and 10 kHz, with a compliance voltage of 100 mV. Magnitude and phase were recorded.

### 2.6 Amplifiers, digitisation, and ENG recording during cystometry

The implanted books were connected to custom-made amplifiers as bipoles, providing two channels per root. The amplifiers are based on commercial instrumentation amplifiers (AD8429- Analog Devices). The amplifier reference was connected to the epidermis via the surgical retractors. The overall amplifier gain was 80 dB [high common-mode rejection ratio (CMRR) of 80 dB up to 5kHz; AD8429 datasheet (Analog Devices, 2017)], and the system bandwidth was 100 Hz–50 kHz. The input-referred noise floor with the inputs shorted was approximately 0.7 μV RMS per channel. This corresponds to a passband input-referred noise density of about 3.27 nV/$\sqrt{Hz}$. The expected amplitudes of the action potentials recorded with a book are very low (typically 1–10 μV for single action potentials and 10–100 μV for compound action potentials).

The amplified and filtered signals were digitized simultaneously using a bank of analog-to-digital converters (NI9222 mounted in cDAQ-9178 by National Instruments, Austin, TX, USA) at a sample rate of 500 kS/s with 16-bit resolution. The high sampling rate (approximately 50 times the Nyquist frequency for the accepted signal bandwidth of 10 kHz) was chosen solely for convenience, and all data were downsampled to a new sample rate of 50 kS/s after recording.

As described above, after electrode implantation and connection to the amplifiers, at least three cystometries were performed per sheep. Cystometry recordings of bladder pressure were time-synchronized with ENG recordings to enable correlation between bladder and ENG responses.

Validation experiments with rhizotomy of S1–S3 distal to the electrode books, first unilaterally (on the right) and then bilaterally, were performed at the end of the experiments in four sheep.

### 2.7 Histological processing and morphometry of sacral roots

Sheep were euthanised by an overdose of pentobarbitone after final cystometry and ENG recordings. The bilateral S1–S3 sacral roots were sectioned cranially and caudally to the respective electrode books and lifted out of their slots. Nerve samples were processed as previously described (Ghnenis et al., 2018). Briefly, they were fixed in 4% paraformaldehyde for a minimum of 48 h before overnight post-fixing in 1% osmium tetroxide (Agar Scientific, AGR1017, UK), then dehydrated through serial concentrations of ethanol, propylene oxide, and increasing concentrations of Araldite CY212 (Agar Scientific, AGR1030) in propylene oxide before overnight embedding in 100% Araldite at 60 ° C. Embedded samples were semi-thin sectioned at 1 μm using an ultramicrotome and stained with methylene blue before slide-scanning at 20 × magnification. The diameter of each scanned section was measured digitally in Fiji ImageJ (Schindelin et al., 2012). Semi-automated morphometry analysis was performed based on previously described methods (Engelmann et al., 2020). Briefly, fascicles were manually cropped from composite images of each section, then contrast-enhanced, auto-local threshold detection was applied, and the number and size of each axon were determined using the “analyse particles” function in Fiji ImageJ. The percentage of axons within the range of diameters expected to carry bladder afferent neural signals [10.1–11.2 μm (Schalow, 2009)] was calculated for each root.

### 2.8 Signal processing

**Figure 2:**
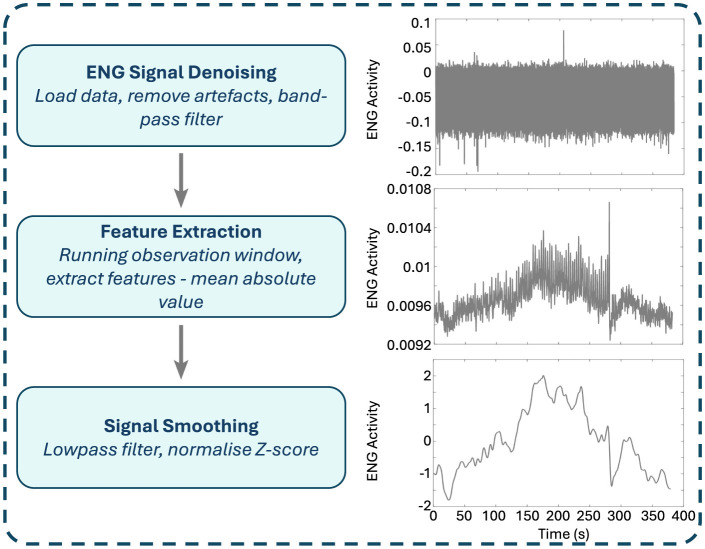
The left shows a flowchart displaying the standard pre-processing steps in MATLAB offline data analysis as follows: artifact rejection, bandpass filtering, running observation window for feature extraction, lowpass filtering and normalization. On the right is an example of an ENG signal recorded during cystometry, shown at each stage of the processing pipeline. The upper trace shows the raw recorded ENG signal after artifact rejection and bandpass filtering, followed by the extracted feature after running observation window, finally the extracted feature after smoothing and normalization.

Data analysis was performed offline using MATLAB (MathWorks, Natick, Massachussetts) to extract bladder fullness information from raw ENG recordings, and Python was used to plot the figures (Metcalfe B. et al., 2021). Standard signal processing, Figure 2, started with artifact rejection, which compared each sample (i.e., individual time point of the ENG trace) with the long-term mean and standard deviation (computed across the full duration of the signal for each channel); samples exceeding threshold T (*Mean*±T*SD) are replaced with the mean. This approach is based on the method described by Jezernik et al. (2001) with a modified threshold (the value of T is given in section 3.4 below) (Metcalfe B. et al., 2021; Jezernik and Sinkjaer, 1998). Next, signals were bandpass-filtered, as ENG signals recorded with extraneural interfaces have an expected bandwidth between 1 and 10 kHz (Raspopovic et al., 2020) and peak spectral power below 3 kHz (Haugland and Hoffer, 1994; Raspopovic et al., 2020). The Running Observation Window (ROW) detected slowly changing features correlated with bladder pressure (Metcalfe B. et al., 2021; Raspopovic et al., 2010). The pipeline concluded with a second-order Butterworth low-pass filter for smoothing and *Z*-score normalization to account for differing baseline signal amplitudes across channels (Metcalfe B. et al., 2021).

Features were extracted to optimize the signal-to-noise ratio (SNR) to augment processing performance (Raspopovic et al., 2020). Standard time domain features are defined in Supplementary Table A1 (Raspopovic et al., 2010; Upshaw and Sinkjaer, 1998; Ramachandran and Siddique, 2019). Pearson's Correlation Coefficient (CC) was used to assess the relationship between each feature and measured bladder pressure.

All the signal processing pipeline hyperparameters, such as artifact removal threshold, bandpass filter cutoff frequencies, window length and overlap, and lowpass filter cutoff frequency, were optimized using a genetic algorithm with six genes and a population size of 10 individuals. While values for these hyperparameters have been reported in the literature, they are generally considered to be rules of thumb or optimized for different animals or tissues. An optimisation algorithm was used to identify the appropriate values for this specific experiment.

Genetic algorithm optimisation was performed using a single representative cystometry intact roots dataset. The resulting hyperparameter set was then fixed and applied identically to all remaining cystometries and animals, without further optimisation, enabling independent evaluation of correlation performance on unseen data. As a control, bladder pressure was randomly permuted and correlations recomputed to assess whether the processing pipeline could produce spurious associations. A population of 10 chromosomes, each comprising a combination of these genes, was evolved for 20–35 iterations of the genetic algorithm until convergence. Six genes were selected as this was the number of hyperparameters being explored. Due to time constraints and computational complexity, the number of individuals and chromosomes was limited to ten. Fitness was assessed by the correlation between the mean of the extracted ENG features (e.g., mean, variance, power, etc.) and the measured bladder pressure. The optimisation algorithm adjusted hyperparameters in the bladder afferent signal processing pipeline to maximize correlation with measured bladder pressure (Raspopovic et al., 2020).

The storage phase of each trial was defined as the period from the start of bladder filling to the time where the catheter was opened. Correlation analyses were conducted separately for this storage phase, as well as across the full trial duration. To quantify uncertainty at the animal level, bootstrap confidence intervals were computed for full-trial correlations. Sensitivity to individual animals was assessed using leave-one-out analyses, in which correlations were recalculated after sequentially excluding each animal from the dataset.

### 2.9 Data analysis

Summary data are reported as *Mean*±*SD* unless otherwise stated. Statistical testing was performed in RStudio Version 1.2.5019 (R Core Team, 2022), GraphPad Prism 10 version 10.2.0 (graphPad Software Inc., San Diego, CA, USA), and SPSS version 28.0.0.0 (190) (IBM SPSS Statistics., Armonk, NY, USA). Data were tested for normality using the Shapiro–Wilk test. The specific statistical tests used are described in the Results.

## 3 Results

### 3.1 Filling cystometries

Maximum bladder volume during recording cystometries was 332 ± 115 ml at 50 cmH_2_O (see Supplementary Figure S3).

### 3.2 Electrode book impedances

Electrode impedances were consistent at between 1 and 3 kΩ with a mean of 1.77 ± 0.50 kΩ at 1kHz (see Supplementary Figure S2 for all results). A significant negative correlation between sacral root diameter and impedance was noted, i.e. impedance values were lower with larger roots [Pearson correlation, *r*(34) = −0.46, *p* = 0.0042] (see Supplementary Figure S1). ENG signal quality was quantified by computing the SNR and power spectral density (PSD) analysis across the 34 cystometries, see Supplementary Figure D1, providing characterization of the raw and preprocessed signals.

### 3.3 Bladder pressure response to electrical stimulation of the sacral roots

**Figure 3:**
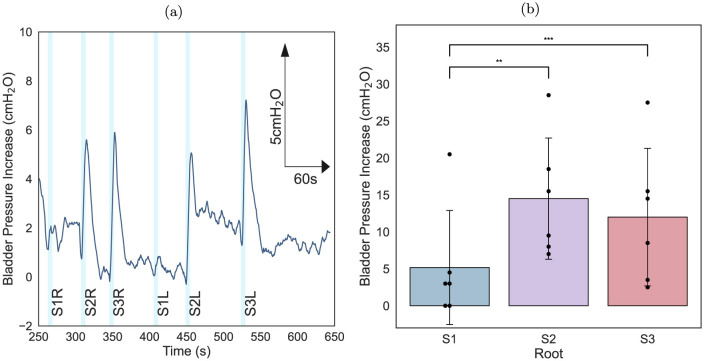
Bladder response to electrical stimulation of the sacral roots. Example trace of bladder pressure is shown in **(a)**, pressure peaks are labeled with sacral root stimulated, note there is a minimal response to stimulation of S1 left and right (S1L, S1R). Averaged across all sheep **(b)**, bladder pressure response to electrical stimulation was significantly greater in the S2 and S3 roots [mixed model *F*_(2, 26)_=11.6, *p* < 0.001, *post hoc* Tukey test ^**^p < 0.01, ^***^p < 0.001].

Bladder pressure increased in response to electrical stimulation of the sacral roots, but the magnitude varied between roots (Figure 3A). Mean bladder pressure increase after stimulation at S1, S2 and S3 was 5.7 ± 7.6, 14.5 ± 8.3, and 12.0 ± 9.3 cmH_2_O, respectively (Figure 3B). A mixed model was used to take account of the variables “sheep," “root," “side of stimulation," and their interactions. Significantly higher bladder pressures were attained in response to stimulation at S2 and S3 roots compared to S1 (*p* < 0.001 and *p* < 0.01, respectively) [*F*_(2, 26)_ = 11.6, *p* < 0.001 and *post hoc* Tukey].

Significant differences in bladder response were also seen between individual sheep [*F*_(5, 26)_ = 18.4, *p* < 0.0001], but there was no significant difference between stimulation of the left and right sides [*F*_(1, 26)_ = 0.5, *p* = 0.5].

### 3.4 Optimized hyperparameters for signal processing pipeline

The results of the genetic algorithm are summarized in Table 1. The *Original* column contains conventional values found in the literature (Jezernik et al., 2000; Sievert et al., 2002), compared against those identified via optimisation.

**Table 1:** Values for hyperparameters used in ENG signal pre-processing based on literature (*Original*) and a genetic algorithm to optimize for correlation to bladder pressure (*Optimized*).

Hyperparameter	Original	Optimized	Effect
Band-pass frequencies	10*Hz*−20*kHz*	75*Hz*−5*kHz*	Reduces noise
Window length (ms)	400	1,000	Improves temporal resolution
Window overlap (%)	0.2%	0.07%	
Artifact removal factor	3.5	5.8	Reduces outliers
Low-pass cut-off frequency (kHz)	10	10	None

These results show that increasing the threshold *T* to 5.8 reduced the rejection rate, as the sample requires a greater deviation from the mean and standard deviation estimates before removal, thereby reducing the risk of losing information correlated with bladder pressure. The band-pass filter frequency range of [75Hz, 5kHz] decreased the appearance of undesired signals, such as out-of-band noise (Raspopovic et al., 2010). The literature states that bladder afferents are typically quite slow at low bladder pressures, and an increase in window length is consistent with this (Bruns et al., 2011).

Mean Absolute Value (MAV) was identified as the most strongly correlated feature with bladder pressure. MAV, therefore, serves as the primary outcome measure for correlating ENG signals with bladder pressure in this study. All neural signals were band-pass filtered from 75 Hz to 5 kHz prior to feature extraction, ensuring that correlations were derived from high-frequency neural activity rather than slow signal envelopes associated with bladder filling. Analysis across all features can be found in the Supplementary Table A2. Similar correlation patterns were observed across multiple amplitude-based features (RMS, power, variance, and standard deviation), indicating that the findings are not dependent on the use of MAV alone.

### 3.5 ENG signal varies with bladder pressure

Detection of ENG in each channel was confirmed by audible response through a loudspeaker to dermal stimulation in the expected dermatome.

**Figure 4:**
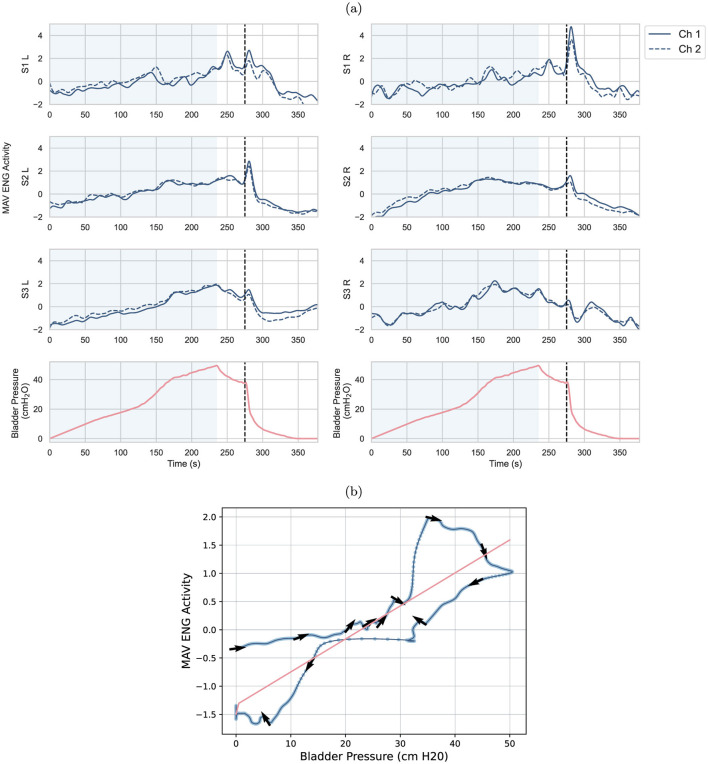
**(a)** Example plot of bladder pressure and ENG activity during filling cystometry, taken across all channels of one sheep during a single cystometry. Bladder pressure is shown in the bottom two panels. The left and right panels are identical and are repeated for ease of comparison with MAV ENG activity in the left and right sacral roots 1, 2, and 3 (S1L–S3R), shown in lines in the six panels above. Channels 1 and 2 in each root are shown as solid and dotted lines, respectively. The shaded area represents the period of bladder filling; the vertical dotted black line marks the opening of the urethral catheter. Features such as peak bladder pressure (occurring at the end of bladder filling; at the end of shaded area), accommodation of the bladder (between the end of bladder filling and urethral catheter opening), urethral opening (vertical dotted line), and bladder emptying (pressure drop to 0) can be identified in MAV activity to different extents in different roots. **(b)** Example phase plot of bladder pressure against sacral root activity in a single channel of a single sheep. The arrows show the direction of travel around the plot. The relationship between bladder pressure and sacral root activity has been fitted with a piecewise linear function.

An example cystometry with bladder pressure and corresponding ENG activity from S1 to S3 roots bilaterally is shown (Figure 4A). During bladder filling (shaded area), bladder pressure (bottom panels) rises characteristically to a peak of 50 cmH_2_O, at which point the infusion pump was turned off. The bladder then accommodates, resulting in a slight drop in pressure. The urinary catheter is then opened (dotted vertical line) and the bladder allowed to drain, with a corresponding rapid drop in pressure to 0 cm H_2_O.

ENG activity (remaining panels of Figure 4A, corresponding to S1–3 roots bilaterally) increases in all roots during bladder filling. Both channels from each root are shown, with a high correlation between them; the average cross-correlation between channels across all experiments was 0.86 ± 0.14. There is variation among roots in the magnitude of this increase, and subjectively, in how closely the ENG activity correlates with bladder pressure. In this example, S3 left (S3L) has the largest change in magnitude during bladder filling, and subjectively, the ENG activity correlates most clearly with bladder pressure. For example, features observed in bladder pressure, such as the gradient changes at around 130 and 170 s, the peak bladder pressure, accommodation, and decreases in pressure as the bladder empties, can all be observed in ENG activity. Some, but not all of these features, can be identified in the other roots displayed here. The MAV ENG activity returns to baseline as bladder pressure returns to 0, except at the S3L root.

Consistent across all except the S3 right (S3R) root is a large peak in ENG activity concurrent with urethral catheter opening and bladder emptying. This feature is observed across all animals.

#### 3.5.1 Non-linear relationship between bladder pressure and ENG activity

Hysteresis is observed in the relationship between bladder pressure and sacral root ENG activity during both bladder filling and emptying. This is illustrated in an example phase plot from one cystometry (Figure 4B), where each point represents the MAV of ENG activity at a given bladder pressure. The lack of return to baseline indicates hysteresis in the system. It could additionally reflect ongoing neural activity that persists after pressure drops, given that the recordings were terminated shortly after voiding.

### 3.6 Validation by sacral root rhizotomy

**Figure 5:**
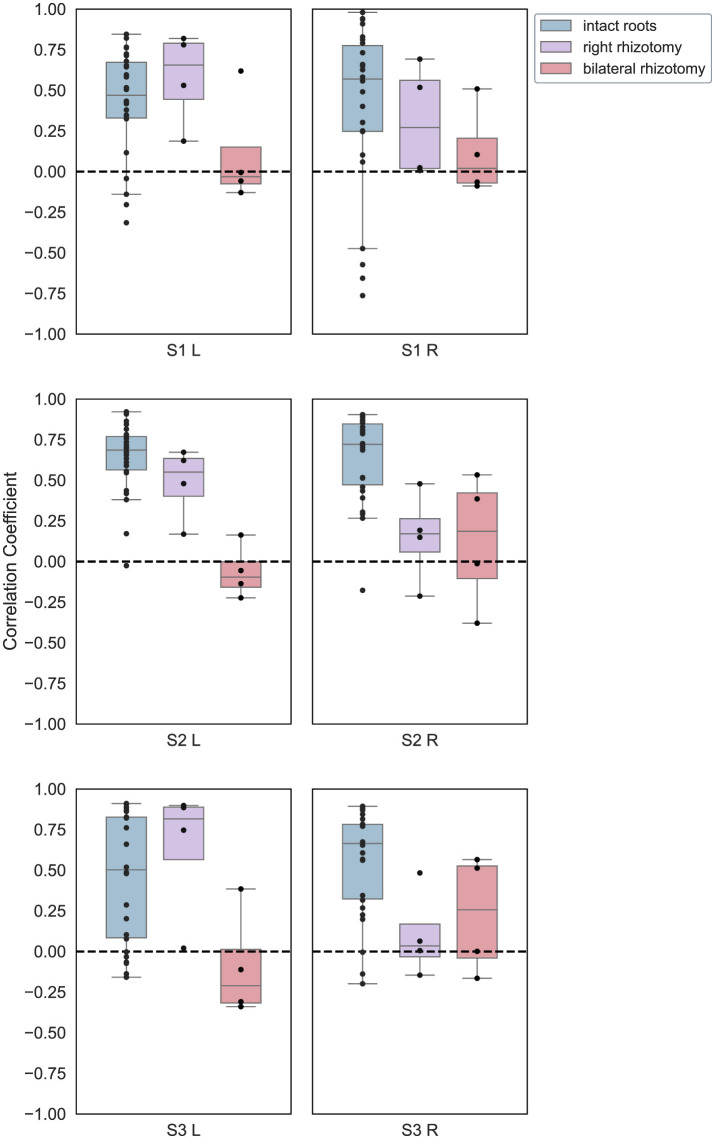
Box plots showing Pearson's correlation coefficient between extracted MAV of the recorded ENG signals and the measured bladder pressure. Plots are shown for sacral roots 1–3 (S1–3) on the left and right (L and R) before rhizotomy (blue) and after rhizotomy of the right (purple), and subsequently bilateral (red) sacral roots. There is a clear reduction in the correlation coefficient after rhizotomy. Note also the increase in correlation coefficient in the left S1 and S3 roots after right rhizotomy.

Filling cystometries and ENG recordings were reported after unilateral (right) and bilateral rhizotomy in four sheep. The selective audible response to dermal stimulation was abolished after distal rhizotomy. Quantification demonstrated that the correlation between bladder pressure and ENG activity is markedly reduced after rhizotomy. After right rhizotomy, correlation is decreased in the right (sectioned) root but persists in the left (intact) root. After bilateral rhizotomy, correlation is lost in both roots (Figure 5). Note that after right rhizotomy, the correlation coefficient in the left S1 and S2 roots (those remaining intact) slightly increases compared to the “intact roots" (no rhizotomy) condition. All correlation analysis are measured across the full time recording; including the catheter-opening and bladder-emptying period.

This is confirmed in statistical analysis (two-way repeated measures ANOVA) across the three datasets—intact roots, right rhizotomy, and bilateral rhizotomy–showing a significant difference in correlation coefficient between ENG and bladder pressure when roots are cut [*F*_(2, 3)_ = 6.2, *p* = 0.034]. There was no statistical significance among channels within the same sheep.

Using fixed optimized parameters, median correlations between MAV extracted from ENG and bladder pressure were similar whether or not the dataset used for optimisation was excluded (Table 2), indicating that results generalize beyond the optimisation data and are not overfitting.

**Table 2:** Median Pearson correlations between sacral root ENG activity and bladder pressure across optimized, validation (excluding the optimisation dataset), and literature-derived parameter sets.

Condition	S1 L	S1 R	S2 L	S2 R	S3 L	S3 R
Optimized (intact, all datasets)	0.470	0.570	0.686	0.722	0.503	0.665
Optimized (intact, excluding optimisation dataset)	0.434	0.558	0.696	0.726	0.519	0.675
Optimized (right rhizotomy)	0.656	0.271	0.551	0.171	0.816	0.035
Optimized (bilateral rhizotomy)	−0.031	0.020	−0.096	0.186	−0.210	0.257
Original (intact)	0.397	0.524	0.614	0.523	0.575	0.525

Genetic algorithm optimisation was performed on a single dataset; all remaining datasets were evaluated using fixed parameters to assess generalization and avoid optimisation bias.

Correlations were reduced following right rhizotomy and were further reduced after bilateral rhizotomy, despite identical processing parameters, indicating that the observed relationships depend on intact afferent pathways rather than the signal processing pipeline or non-bladder-related signals. As a control, bladder pressure was randomly permuted to disrupt temporal structure, resulting in near-zero correlations across all roots (0.034 ± 0.023). This confirms that the observed correlations are not produced by the signal processing pipeline.

Comparable root patterns were present when using literature-derived parameters prior to optimisation [Original parameters in Table 1, containing conventional values found in the literature (Jezernik et al., 2000; Sievert et al., 2002)], demonstrating that optimisation does not generate the observed relationships.

Correlations during the storage phase were comparable to those computed over the full trial (Table 3), with only some differences across roots. S2 activity showed the least variation in correlation with bladder pressure between storage phase and the full trial, consistent with the representative ENG recordings. Despite the presence of transient artifacts associated with catheter opening, correlations in the full-trial remained consistent with those measure in the storage phase, indicating that the observed relationships are not dominated by these non-neural or mechanically induced signals.

**Table 3:** Comparison of correlation to bladder pressure for full-trial data (including bladder emptying) and the storage phase only.

Root	Full trial	Storage phase	95% CI	LOOCV range
S1L	0.46 ± 0.23	0.55 ± 0.26	0.02, 0.65	0.55–0.58
S1R	0.57 ± 0.26	0.66 ± 0.22	−0.18, 0.80	0.57–0.65
S2L	0.64 ± 0.13	0.64 ± 0.29	0.44, 0.76	0.67–0.72
S2R	0.64 ± 0.22	0.59 ± 0.32	0.55, 0.85	0.716–0.717
S3L	0.51 ± 0.32	0.68 ± 0.25	0.00, 0.86	0.10–0.66
S3R	0.58 ± 0.25	0.59 ± 0.30	0.29, 0.82	0.59–0.66

All values displayed as Mean ± STD. For added robustness, confidence intervals computed via animal-level bootstrap (*n* = 6), and leave-one-out cross validation (LOOCV) tests were performed.

In the intact root condition, correlation between MAV extracted from ENG and bladder pressure were consistently highest in the S2 roots (Table 3). Animal-level bootstrap confidence intervals in the full trial indicated variability across animals, but were narrowest for the S2 roots, suggesting greater consistency. To assess robustness, leave-one-out analyses were performed between animals; median correlations remained stable across exclusions, indicating that results were not driven by any single animal. These analyses indicate that, despite the small sample size and inter-individual variability, the main findings are robust and not driven by any single animal.

### 3.7 Distribution of afferent bladder signal between the sacral roots

The magnitude of cross-correlation of ENG to bladder pressure was assessed for each root in each sheep (*n* = 6 sheep). A mixed model was used to take account of the variables “sheep," “root," “side of recording," and their interactions. A significant effect of sheep [*F*_(5, 137)_ = 12.3, *p* < 0.001], root [*F*_(2, 137)_ = 9.8, *p* < 0.001] and their interaction [*F*_(10, 137)_ = 6.6, *p* < 0.001] was seen. The side of the recording did not significantly affect the correlation.

**Figure 6:**
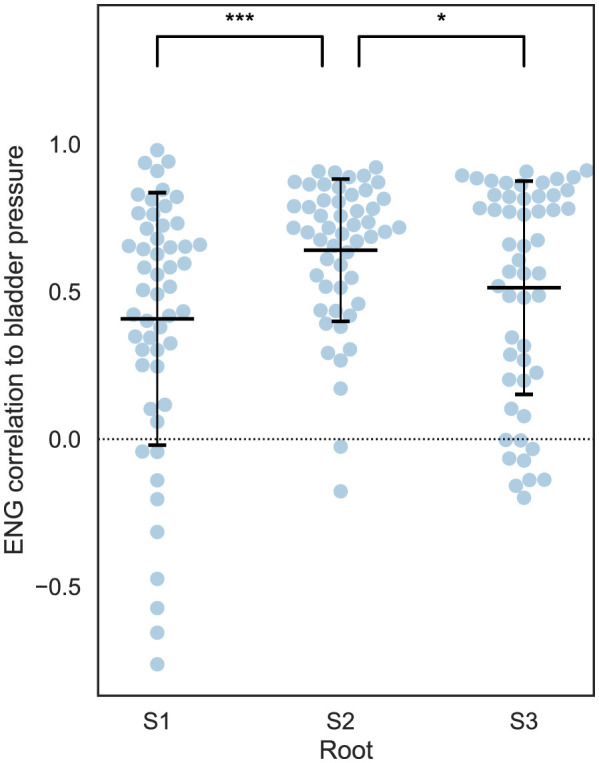
Correlation of afferent ENG signal to bladder pressure is shown in each sacral root (S1, S2, S3). Despite wide variation within each root, on average, the correlation between ENG and bladder pressure is significantly higher in the S2 root compared to S1 and S3 [mixed model [*F*_(2, 137)_ = 9.8, *p* < 0.001, *post hoc* Tukey test ^*^p < 0.05, ^***^p < 0.001]. Each dot represents a single cystometry experiment.

S2 had the highest overall ENG correlation to bladder pressure at 0.64 ± 0.13, significantly higher than S3 at 0.50 ± 0.25 (*post-hoc* Tukey test, *p* = 0.044) and S1 at 0.41 ± 0.37 (*p* < 0.001; Figure 6). Note that, as implied by the statistical results above, the correlation between root and afferent signal was variable between individual sheep. For example, the correlation coefficient between S1 and and bladder pressure was 0.70 in sheep 1 and 2, and was 0.72 between S3 and bladder pressure in sheep 4, 5, and 6.

### 3.8 Fiber size distribution in the sacral roots

**Figure 7:**
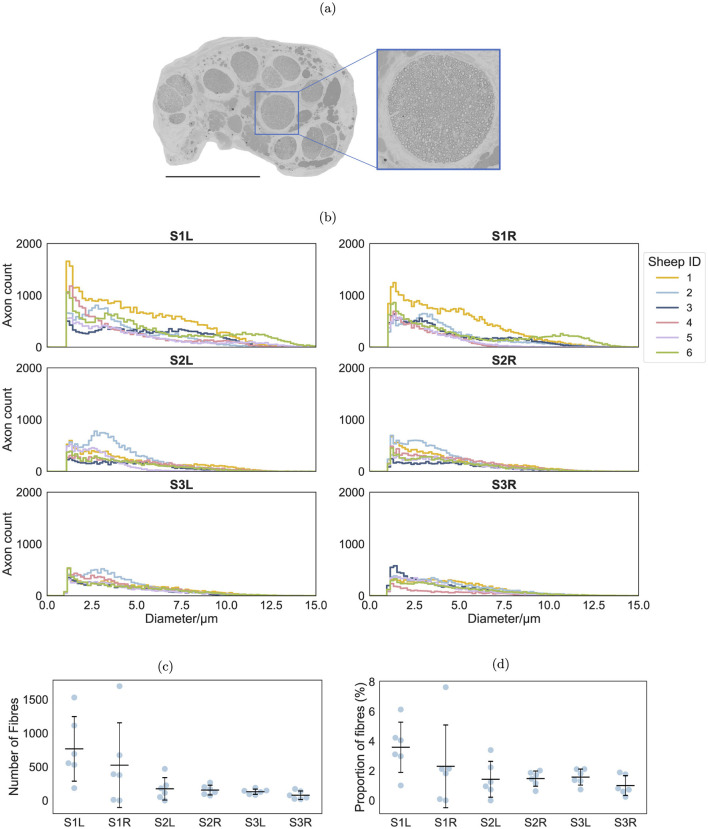
Fiber size withing the sacral roots. Photomicrographs **(a)** show fasciculation within the sacral root, with a spread of fiber diameters in each fasciculation. The black line is scale bar of 1mm. Fiber diameters from all sheep are displayed for each root **(b)**, each panel represents 1 root as labeled, colored lines represent each sheep). The absolute number of expected bladder afferent fibers **(c)** and proportion of expected bladder afferent fibers **(d)** within this range are shown. No significant difference between roots is identified.

During surgery, the S3 roots were smaller than the S2 roots, which were smaller than the S1 roots. On histological examination, the diameter of S1 was 2.7 ± 0.8 mm, of S2 was 2.0 ± 0.5 mm, and of S3 was 1.4 ± 0.6 mm. The average for all roots was 2.1 ± 0.9 mm. The roots were variably fasciculated, and a range of fiber sizes could be appreciated microscopically as expected (see Figure 7A). The distribution of fiber diameters within each sacral root of each sheep was calculated and is summarized as histograms (see Figure 7B). Fiber sizes ranged between 1 and 15μm, with S1 roots having a larger number of fibers, but in general, a similar distribution was seen across all roots. There is no significant difference in the absolute number (Figure 7C) or proportion (Figure 7D) of fibers in the range 10.1–11.2μm (the expected diameter range of bladder afferent fibers (Schalow, 2009).

### 3.9 Comparing bladder pressure response to stimulation, ENG correlation to bladder pressure, and fiber distribution

In four of the six sheep, the sacral root whose electrical stimulation caused the highest bladder pressure increase was the same as the sacral root with the highest correlation of ENG activity to bladder pressure. This was in one of the S2L, S2R, S3L, and S3R roots, respectively.

An exploratory comparison between the factors reported in this paper was therefore conducted: namely, a comparison between (i) bladder pressure response to stimulation of the individual sacral roots; (ii) ENG correlation to bladder pressure within each sacral root; and (iii) afferent fiber distribution within each sacral root. Pearson's correlation coefficient results are shown in Table 4. There is no clear correlation among these factors, although a very weak positive correlation exists between the bladder pressure response to stimulation and the ENG correlation with bladder pressure. Statistical analysis was not further pursued due to the low statistical power and exploratory nature of this comparison.

**Table 4:** Exploratory pairwise Pearson's correlation coefficient between the three datasets obtained from each sheep in this study: ENG correlation to bladder pressure (“*ENG*)", bladder pressure response to electrical stimulation of the sacral roots (“*stim*"), and fiber size distribution within the sacral roots (“*fiber*").

Root	ENG and Stim.	ENG and fiber	Fiber and Stim.
S1L	−0.24^*^	0.44	0.34^*^
S1R	0.88	−0.86	−0.70
S2L	0.035	0.35	−0.20
S2R	0.28	−0.55	−0.30
S3L	0.43	−0.25	−0.056
S3R	0.32	−0.31	−0.27
Avg.	0.28	−0.11	−0.20

^*^No stimulation tests for S1L root of sheep 1.

## 4 Discussion

This study has demonstrated the feasibility of concurrently recording bladder afferent ENG from multiple sacral roots using extra-dural electrode books. To the authors' knowledge this is the first demonstration of recording bladder afferent signal from this number of sacral roots concurrently, and is the first assessment of the distribution of bladder afferent activity across the sacral roots. Recording bladder afferent signals is the first step toward a closed-loop system for the potential treatment of DSD or for restoring bladder function. The bladder pressure response to stimulation across the same sacral roots was further assessed, allowing for a direct comparison of stimulation and afferent data. In addition, morphometry data were provided to show the distribution of fiber diameters within each root.

### 4.1 Correlation between ENG activity and bladder pressure

Overall, a good correlation between recorded ENG and bladder pressure has been shown using this extra-dural electrode book method. Recorded correlation coefficients between bladder pressure and ENG are comparable to those reported in the literature. At 0.64 ± 0.13, the reported correlation is lower than that reported from multi-unit activity recorded from sacral dorsal root ganglions using microelectrode arrays in cats (0.81 ± 0.13), as might be expected from intraneural recordings in a smaller animal and nerve (Ross et al., 2018). However, the reported correlation is higher than that found in pigs using a unilateral nerve cuff on S3 (0.45 ± 0.27) (Jezernik et al., 2000). Recordings were also more reliable; bladder afferent signals were obtained in all six sheep and across repeat cystometries, compared to previously reported correlation of ENG and bladder pressure in just three out of seven pigs (Jezernik et al., 2000) and two of six cats with a nerve cuff on a sacral root (Jezernik et al., 2001).

### 4.2 Validity of recordings

In line with previous reports using extra-dural, less-invasive recording devices such as nerve cuffs, the magnitude of the ENG variance detected was very small (Jezernik et al., 2000; Metcalfe et al., 2020). However, identification of hysteresis between bladder pressure and bladder afferent ENG provides confidence in the recordings. This effect has been identified previously in the literature and is believed to result from a combination of the intrinsic elastic and mechanical properties of the bladder wall, and potentially from the membrane properties of mechanoreceptors themselves (Jezernik et al., 2000; Bruns et al., 2011; Ross et al., 2016; Metcalfe et al., 2020).

The validity of the recordings is further corroborated by a significant decrease in ENG correlation with bladder pressure following rhizotomy of the implanted sacral roots, as expected. Interestingly, there was a slight increase in ENG correlation to bladder pressure in the intact left S1 and S3 roots after right rhizotomy. The dataset is small (*n* = 4), but the high correlation between repeats within animals (Pearson's coefficient >0.6) gives reasonable statistical power. Further data would be needed to confirm whether there is a genuine increase in bladder afferent signal through the contralateral roots after unilateral rhizotomy, or whether this is an artifact of disrupting the expected network of spinal interneurons and arising closed-loop lumbosacral circuits in this region. These neural circuits play a role in coordinating the external and internal urethral sphincters and detrusor muscles (for a review, see Karnup, 2021) and serve as a reminder of the complexity of the physiological system that a closed-loop control device for urination interacts with.

Interestingly, a large peak in ENG activity is seen consistently and is associated with urethral catheter opening and bladder emptying. It is likely that upon opening the catheter, there was some physical movement and, therefore, urethral stimulation. It is possible, therefore, that the large peak reflects more intense stimulation compared to bladder filling or, indeed, that the urethra has greater afferent innervation. It is also possible that some of this recorded signal is a reflex efferent signal to the urethra. However, separating specific afferent and efferent signals has been challenging due to the limited number of channels in the electrode books. Further advanced signal processing is beyond the scope of this paper. Longitudinal array cuff electrodes can be used to extract temporal information of signals, such as signal velocity, through velocity selective recording (VSR), enabling the separation of afferent and efferent activity (Metcalfe B. W. et al., 2021; Taylor et al., 2012). Using an array cuff electrode with more channels in future experiments may therefore provide a means to better discriminate afferent and efferent signals.

The storage phase was analyzed separately to confirm that the observed correlations were not driven by non-bladder-specific signals associated with catheter opening. Bladder emptying was not analyzed as a separate phase, as it was not a primary focus of this study and is dominated by transient, non-stationary activity associated with catheter opening and voiding. During the storage phase, the absence of the transient artifact associated with catheter opening provides a cleaner assessment of bladder-related neural activity.

Correlations observed during the storage phase were comparable to those obtained over the full trial, indicating that the main findings are not dependent on inclusion of the voiding period. In particular, the S2 root demonstrated consistently high correlations with reduced variability across animals in both analyses. This agreement between full-trial and storage-only results supports the conclusion that the reported relationships reflect bladder afferent activity during filling, rather than being driven by events specific to voiding. Further, this indicates that the discrepancies between recordings and correlation analysis are not driven by the bladder-emptying period.

Although a reference electrode connected to surgical retractors may introduce common mode noise, this noise would be expected to affect all conditions similarly. The presence of correlations across all roots in the intact root case and their loss following bilateral rhizotomy suggests that common mode contributions are unlikely to account for the observed relationships. Further cross-correlation analysis of band-pass filtered ENG signals showed low and variable zero-lag correlation between channel pairs (|*r*| < 0.5; Mean ± SD: −0.15 ± 0.02), indicating that recordings are not dominated by common-mode noise.

### 4.3 Distribution of afferent signal

Recording from multiple sacral roots concurrently (S1–3 bilaterally) in this study has shown that afferent bladder signals are present in all these roots in sheep. On average, the S2 root carries the predominant afferent signal, but this varies substantially across individuals.

Given the low SNR of book-electrode ENG and the variance between individuals, these findings should be used as a foundation for future exploration to determine in a larger population that may be more representative. This is compatible with the pilot report in one sheep instrumented in the same way as this study (although in this sheep S1 and S2 had the highest increase in ENG) (Metcalfe B. et al., 2021), and is the only other report the authors are aware of in sheep. In pigs, there is a suggestion that S3 may have more bladder afferent signal than S2 (Jezernik et al., 2000) and in cats that S1 may have more bladder afferent signal than S2 (Jezernik et al., 2001)—but all roots are not tested in these species. As outlined in the introduction, anatomical studies have suggested wide variation across species and individuals, with the S2 and S3 nerve roots likely to carry afferent bladder signals in humans.

### 4.4 Relationship between efferent and afferent activity

For a closed-loop neuroprosthesis to control urinary incontinence, it would be logical to implant recording electrodes on the root(s) with the greatest afferent innervation and stimulation electrodes on the root(s) with the greatest efferent innervation. It would be clinically convenient to be able to assess which root(s) are likely to have the highest afferent signal (greatest correlation of ENG to bladder pressure), and an outstanding question, therefore, remains as to whether the root with the highest efferent innervation to the bladder (as determined by bladder pressure response to electrical stimulation of the sacral roots) correlates with the root with the highest afferent innervation.

This relationship appeared true for four of the six sheep in this study. This is an interesting finding and is consistent with our first hypothesis, but the sample size is too small to conclude whether this is representative and, therefore, whether the majority of the population might have this relationship between efferent and afferent activity. This is particularly true, as there is no convincing correlation in this study population between ENG activity and bladder pressure, or between bladder pressure response to stimulation and ENG activity (the Pearson correlation coefficient between these two factors is low across all sacral roots).

This study shows little difference in fiber-diameter distribution across the sacral roots, providing no evidence to support our second hypothesis. A limitation of morphometry is that it does not give any information on fiber type (beyond size), i.e., whether the fiber is afferent or efferent, and what it innervates. Myelinated afferent cutaneous sensory fibers and motorneurons are reported to have a comparable diameter to bladder afferent fibers of around 12 μm (Schalow, 2009), so this may be confounding the interpretation of morphometry. Indeed, the number of bladder afferent fibers is likely to be small, with estimates that less than 3% of sacral dorsal root ganglia innervate the lower urinary tract in cats (de Groat and Yoshimura, 2009). This may, therefore, be below the limit for detectable variation between roots. Given this, the lack of correlation to fiber distribution is unsurprising. In future studies, anatomical neuronal tracing experiments could be performed to determine the exact number of bladder afferent fibers and their distribution, which correlated to electrophysiology data.

### 4.5 Relevance for clinical translation

It is encouraging for clinical translation that robust detection of bladder afferent signals is possible in a large-animal model using extra-dural electrode books. Recording of bladder afferent signal is more commonly reported from more invasive intraneural microelectrode arrays in smaller animals (e.g., cats) (Bruns et al., 2011; Ouyang et al., 2019, 2022; Lubba et al., 2021; Ross et al., 2018). Despite the development of more flexible variants of these arrays (Sperry et al., 2021), these implants still cause significant tissue reaction and inflammation *in vivo*. The electrode books in this study, by contrast, are comparable to those already used clinically for long-term implantation in SARS and are therefore known to be safe.

The sheep model used in this study is relevant to the translation of device development to humans. Sheep are a comparable weight to humans with a comparable sacral vertebral canal diameter; ~20mm in humans (Bagheri and Govsa, 2019), and ~10 mm in sheep based on experience. Sheep peripheral nerves, like humans, are fasciculated and have similar dimensions with similar myelinated afferent nerve conduction velocities (Alvites et al., 2021; Schalow, 2009). The logistical and engineering challenges in acute recordings are therefore similar between humans and sheep for sensory nerve recordings at the sacral roots.

Given the highly individual distribution of afferent and efferent activity across sacral roots, and the apparently variable relationship between these activities within each root, it may be necessary to design implants capable of recording bladder afferent information from multiple roots. Recording from multiple roots concurrently also opens the possibility that ENG from individual roots could be integrated together to provide a more robust and reliable detection of bladder afferent signal. Although this has not been necessary for this dataset, it may be valuable in chronic and clinical settings, where the signal-to-noise ratio is expected to be lower (due to movement-related ENG), and individual electrodes can be obscured by fibrotic tissue (Donaldson et al., 2023). Alternatively, if it is clinically preferable or necessary to implant on only one root, choosing the single root with the largest bladder response to electrical stimulation (presumed to have the highest bladder efferent activity) remains the best option, based on the limited data in sheep. In this circumstance, a longer multi-electrode nerve cuff and velocity selective recording techniques are expected to improve sensitivity and discrimination of signal type, as well as afferent and efferent signal (Taylor et al., 2011; Metcalfe et al., 2020; Donaldson et al., 2023).

## 5 Conclusions

This is the first study to demonstrate the feasibility of concurrently recording bladder afferent ENG from multiple sacral roots using extra-dural electrode books with a design currently used for chronic clinical implantation. It is also the first study to assess the distribution of bladder afferent activity across roots.

Assessing the distribution of bladder afferent signals has identified significant variation among animals in which sacral roots exhibited the largest afferent and efferent responses. This has important ramifications for the design a closed-loop control neuroprosthesis as it suggests a clinical decision may need to be made in each patient about which root to implant on, or implies multiple sacral roots must be implanted in every patient.

The electrode books used in this study are comparable to those employed for chronic implantation in clinical settings. In addition, sheep are a large animal model of similar size to humans and, therefore, exhibit typical signal-to-noise limitations, as in humans. This ensures that these findings hold significance for clinical translation and the development of a neuroprosthetic device for human patients.

## Data Availability

The raw data supporting the conclusions of this article will be made available by the authors, without undue reservation.

## Supplementary material

Supplementary Figure S1 Relationship between electrode impedance and sacral root diameter. A significant negative correlation was seen between sacral root diameter and impedance [Pearson correlation, *r*(34) = −0.46, *p* = 0.0042].

Supplementary Figure S2 Violin plot showing summary of electrode impedances in all sacral roots at all frequencies measured.

Supplementary Figure S3 Plot of maximum bladder volume during each recording cystometry for all six sheep used in experiments. Error bars represent mean ± SD.
