# Supplementary material for: Distribution of bladder afferent activity across the sacral roots in sheep shows marked individual variation: implications for neuroprosthesis design
Source: Front Neurosci. 2026 Jun 12;20:1857570. doi: 10.3389/fnins.2026.1857570 (PMC13303735; doi:10.3389/fnins.2026.1857570)
Supplement: Supplementary Figure S1 — Relationship between electrode impedance and sacral root diameter. A significant negative correlation was seen between sacral root diameter and impedance [Pearson correlation, r(34) = −0.46, p = 0.0042]. [file Supplementary_file_1.pdf]

Appendix A. Running Observation Window (ROW) time domain feature extraction methods

Table A1: Standard time domain features that can be extracted from ENG signals

Feature	Equation
Root Mean Square (RMS)	$\sqrt{\frac{1}{n}\sum_{i=1}^{n} x_i^2}$
Power	$\frac{1}{n}\sum_{i=1}^{n} x_i^2$
Variance	$\frac{\sum(x_i-\mu)^2}{n-1}$
Mean	$\frac{\sum x_i}{n}$
Mean Absolute Value (MAV)	$\frac{\sum \lvert x_i \rvert}{n}$
Standard Deviation	$\sqrt{\frac{\sum(x_i-\mu)^2}{n}}$
Autocorrelation	$\frac{\sum_{i=k+1}^{n}(x_i-\mu)(x_{i-k}-\mu)}{\sum_{i=1}^{n}(x_i-\mu)^2}$
Skewness	$\frac{\sum_i^n (x_i-\mu)^3}{(n-1)\times\sigma^3}$
Crest Factor	$\frac{\lvert x_{peak} \rvert}{x_{rms}}$
Impulse Factor	$\frac{\max(\lvert x \rvert)}{\frac{1}{n}\sum_{i=1}^{n} \lvert x[n] \rvert}$
Total Harmonic Distortion (THD)	$\frac{x_{rms}}{x_1}$
Kurtosis	$\frac{1}{n}\sum_{i=1}^{n}\left(\frac{x_i-\mu}{\sigma}\right)^4$

RMS, power, variance, standard deviation, and MAV all quantify signal amplitude or energy and therefore produce similar results (Table A2), as expected. These features are mathematically related and represent equivalent measures of broadband signal energy. The consistency across these metrics indicates that the observed correlations with bladder pressure are not dependent on the specific choice of amplitude-based feature.

This analysis demonstrates that the measured correlations with bladder pressure are specific to amplitude-related features derived from high-frequency neural activity. These correlations are reduced following bilateral rhizotomy, confirming that they depend on intact afferent pathways rather than arising from non-bladder-related signal properties. In contrast, higher-order statistical and shape-based features do not show meaningful associations, supporting the observed relationships.

Table A2: Average correlation to bladder pressure across all tested features for all animals.

Feature	Intact Roots	Right Rhizotomy	Bilateral Rhizotomy
RMS	0.553	0.430	-0.0554
Power	0.550	0.428	-0.0548
Variance	0.550	0.428	-0.0548
Mean	-0.0127	-0.0167	0.00547
MAV	0.571	0.433	-0.0310
Standard Deviation	0.553	0.430	-0.0554
Autocorrelation	0.320	0.0397	-0.0316
Skewness	-0.0542	0.0956	0.0285
Crest Factor	0.00452	0.00539	-0.0070
Impulse Factor	0.008	0.00956	-0.0077
THD	0.0408	0.055	-0.0088
Kurtosis	0.0671	0.0476	0.00504

Appendix B. Filling Cystometries

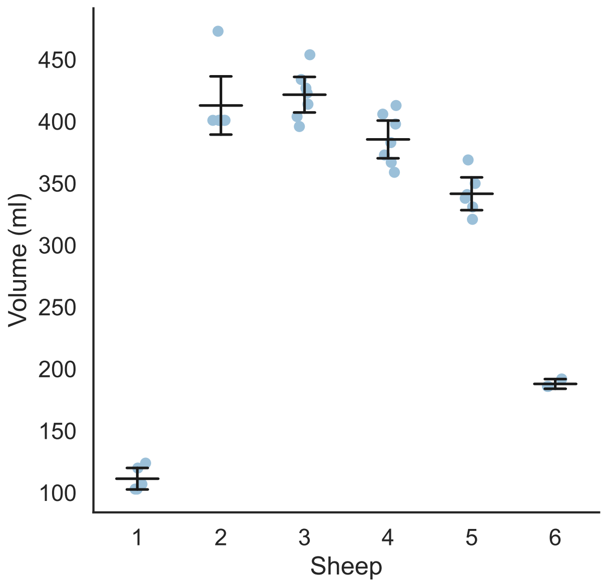

Figure B1: Plot of maximum bladder volume during each recording cystometry for all six sheep used in experiments. Error bars represent mean ± SD.

Appendix C. Electrode impedances

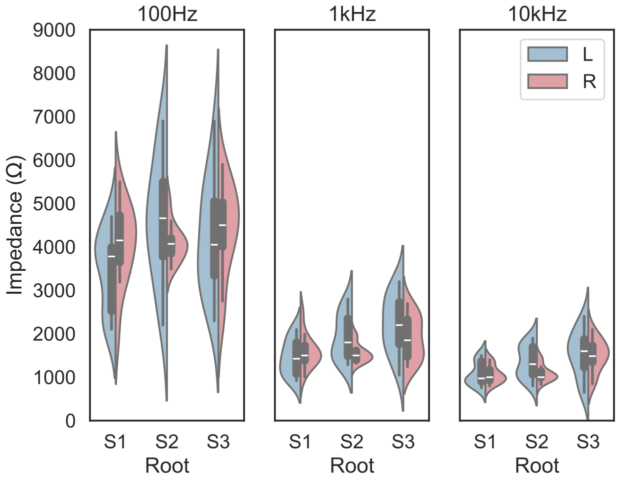

Figure C1: Violin plot showing summary of electrode impedances in all sacral roots at all frequencies measured.

Appendix D. ENG signal quality validation

To quantify the ENG signal quality, SNR was computed across the 34 cystometries before and after bandpass filtering and artefact rejection (signal preprocessing). As expected for extraneural book-electrode recordings, SNR values were low and showed

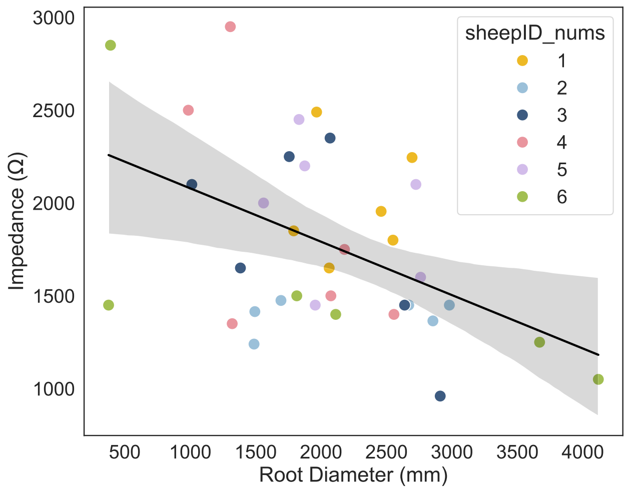

Figure C2: Relationship between electrode impedance and sacral root diameter. A significant negative correlation was seen between sacral root diameter and impedance [Pearson correlation, r(34) = -0.46, p=0.0042]

variability between animals (-14.3 ± 5.7 dB). Such low SNR values are consistent with previously reported peripheral nerve recordings for extraneural electrode [**?**].

Despite the low absolute SNR, further analysis shows that correlations with bladder pressure are consistently observed in intact roots, are preserved across independent datasets, and are lost following bilateral rhizotomy. Additionally, correlations are absent in temporally shuffled control data. This indicates that the extracted features capture physiologically relevant information not attributable to noise alone.

Power spectral density (PSD) analysis for a raw intact roots dataset (Figure D1) shows broadband activity within the expected ENG frequency range. While the spectral profile is dominated by noise-like components, discrete peaks and deviations from the baseline are present, suggesting structured signal content embedded within the broadband noise.

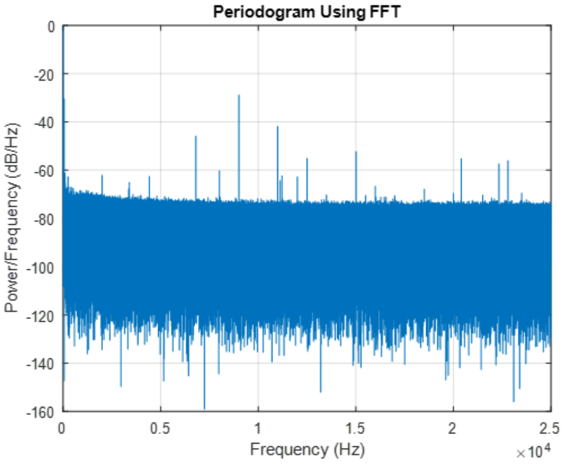

Figure D1: Power spectral density (PSD) analysis for a single dataset from a single animal pre-denoising.
